# UV-C Light Intervention as a Barrier against Airborne Transmission of SARS-CoV-2

**DOI:** 10.3390/v16010089

**Published:** 2024-01-05

**Authors:** Izabela Ragan, Jessie Perez, Wilson Davenport, Lindsay Hartson, Branden Doyle

**Affiliations:** 1 Department of Biomedical Science, Colorado State University, Fort Collins, CO 80521, USA; 2 Violett Inc., Gig Harbor, WA 98332, USAbranden@violett.com (B.D.); 3 Department of Microbiology, Immunology & Pathology, Colorado State University, Fort Collins, CO 80521, USA

**Keywords:** SARS-CoV-2, transmission, respiratory, viruses, healthcare, sterilization, air, hamster model, UV-C light, inactivation

## Abstract

Background: SARS-CoV-2 continues to impact human health globally, with airborne transmission being a significant mode of transmission. In addition to tools like vaccination and testing, countermeasures that reduce viral spread in indoor settings are critical. This study aims to assess the efficacy of UV-C light, utilizing the Violett sterilization device, as a countermeasure against airborne transmission of SARS-CoV-2 in the highly susceptible Golden Syrian hamster model. Methods: Two cohorts of naïve hamsters were subjected to airborne transmission from experimentally infected hamsters; one cohort was exposed to air treated with UV-C sterilization, while the other cohort was exposed to untreated air. Results: Treatment of air with UV-C light prevented the airborne transmission of SARS-CoV-2 from the experimentally exposed hamster to naïve hamsters. Notably, this protection was sustained over a multi-day exposure period during peak viral shedding by hamsters. Conclusions: These findings demonstrate the efficacy of the UV-C light to mitigate against airborne SARS-CoV-2 transmission. As variants continue to emerge, UV-C light holds promise as a tool for reducing infections in diverse indoor settings, ranging from healthcare facilities to households. This study reinforces the urgency of implementing innovative methods to reduce airborne disease transmission and safeguard public health against emerging biological threats.

## 1. Introduction

The SARS-CoV-2 global pandemic has killed over 6.9 million people and new variants are still emerging [1]. Over the course of the last three years, it has become clear that the primary means of transmission from infected individuals is respiratory, including respiratory droplets and aerosols [2]. In response, several strategies have been implemented to reduce airborne transmission and mitigate infection risk including stringent hygiene practices, social distancing, wearing masks, improving indoor ventilation, moving gatherings to outdoor areas, altering indoor air humidity levels, and vaccination [3,4,5,6,7]. Importantly, SARS-CoV-2 infection can lead to severe immunopathogenesis, including a hyper-inflammatory response and cytokine storm-like syndrome, which is innate immune activation that can exacerbate disease severity [8,9]. This highlights the need for effective mitigation strategies against airborne transmission. While social distancing guidelines were implemented based on the risk of droplet transmission, the potential for transmission beyond the defined distances is significant [10]. Indoor settings such as healthcare facilities, restaurants and workplaces are at risk of long-distance (greater than 2 m) aerosol transmission [11,12]. Aerosols containing SARS-CoV-2 viral particles are stable for hours [13];. therefore, indoor masking and vaccination were used frequently during the pandemic. Mask-wearing has decreased as we transition to daily life post-pandemic [14] and vaccine hesitancy toward COVID-19 boosters is increasing in some countries [15]. Therefore, new mitigation strategies beyond social distancing are needed to address aerosol transmission.

Within this context, a number of studies have highlighted the potential for UV-C light as a means of mitigating the transmission of SARS-CoV-2 in indoor settings. UV-C can be used for sanitization of surfaces and sterilization of aerosols and offers a safer alternative to chemical-based treatments like hydrogen peroxide vaporization [16]. Noteworthy is the finding that UV-C light doses of 34.9–52.5 mJ/cm^2^ can inactivate SARS-CoV-2 on surfaces [17,18,19], while 21.4 mJ/cm^2^ dosing can inactivate SARS-CoV-2 in the air [20] over brief exposure durations. The current study extends these findings by demonstrating the capacity of UV-C light to provide long-term protection from airborne SARS-CoV-2 transmission over an extended multiday period. We employ a susceptible hamster model to demonstrate the effectiveness of UV-C light as a means of inactivating SARS-CoV-2 in indoor settings and safeguarding against airborne transmission.

## 2. Materials and Methods

### 2.1. Violett Air Sterilization Device

The Violett sterilization device was designed and developed by Violett Inc. The device uses a fan to draw air through a HEPA filter into a UV-C light chamber. The selected UV-C LEDs in the Violett device produce a peak germicidal wavelength of 265 nm with very tight emittance bands (+/−5 nm) (Bolb S6060-DR250-W275-P100-V6.5). The UV-C light chamber is contained within an external housing, and inlets and outlets to the chamber are minimized. This allows UV-C photons to remain inside the chamber and available to kill airborne pathogens, and not leave the device. UV-C light is known to be hazardous to the skin and eyes [21], thus it is important that the UV-C light remain inside the device. This was independently validated through 3rd party testing (Intertek Laboratories) Additionally, the Violett device is safe to operate in the presence of animals and animal welfare staff. The inner portion of the UV-C chamber is coated with highly reflective material to minimize photon loss to chamber materials and to increase germicidal efficiency (Porex PMR20 AB). The UV-C light chamber is designed such that its unique shape, air inlet, and air outlet locations create a cyclonic path for airborne particles to travel through. This cyclonic path ensures that airborne particles such as viruses spend an increased amount of time in the UV-C light field, while the combination of the reflective material coating the chamber and the high intensity of the UV-C emitter array maximize UV-C dosing of airborne pathogens. The high UV-C dosing allows for pathogens that make it through the HEPA filter to be dosed and inactivated prior to leaving the UV-C chamber and the Violett device. Together, these features distinguish the Violett device from other commercially available devices and provide a novel experimental approach for this work.

A control device was used to pull air through the unit without treatment by HEPA filtration and UV-C LEDs. The use of a fan without HEPA filtration or UV-C light in the control device replicated changes in air currents produced by the fan and ducting in the Violett device. Both Violett and control devices were connected to the animal housing with six feet of 3-inch diameter duct hose (Bio-R-Vac hose McMaster PN 56675 K48). All other aspects of animal housing were kept constant between the control and experimental groups. 

### 2.2. Animals

We used a total of 27 male Golden Syrian hamsters (*Mesocricetus auratus*) who were 7–9 weeks old. All hamsters were held at Colorado State University in the Association for Assessment and Accreditation of Laboratory Animal Care (AAALAC) International accredited animal facilities (Accreditation #000834). Animal testing and research received ethical approval from the Institutional Animal Care and Use Committee (IACUC). Hamsters were acquired from Charles River Laboratories (Wilmington, MA, USA) and were maintained in a Biosafety Level-3 (BSL-3) animal facility at the Regional Biocontainment Lab at Colorado State University during the study.

### 2.3. Study Design

**Figure 1:**
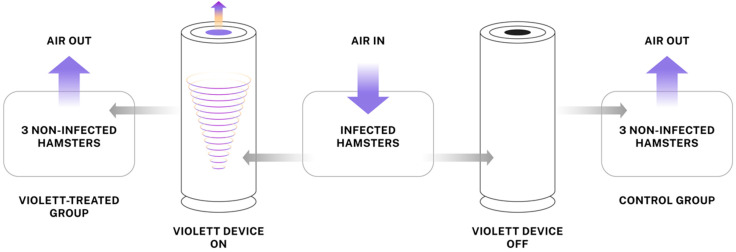
Aerosol SARS-CoV-2 transmission system using UV-C light to block transmission. The Violett device contains a HEPA filter and UV-C LEDs to sterilize air that may be carrying SARS-CoV-2 viral particles. An aerosol transmission system to test the effectiveness of the device was designed by modifying hamster cages to enable direct airflow from infected hamsters to non-infected hamsters.

**Figure 2:**
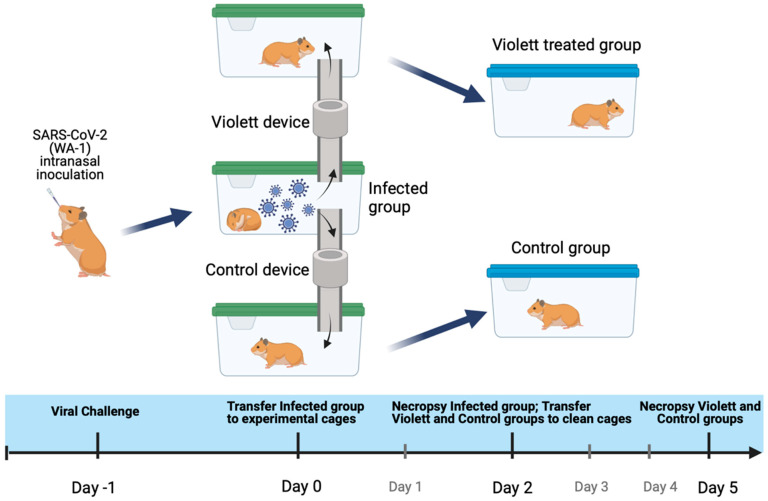
Experimental airborne transmission of SARS-CoV-2 using a hamster model. Hamsters were intranasally challenged with SARS-CoV-2 and transferred to the center cage after 24 h. Violett devices were placed between the infected hamster cage and non-infected hamster cages to treat air moving from the infected hamster cage into the periphery cages. The non-infected hamsters were exposed to air circulating from the Infected group cage over a 48-hour period. After two days, the hamsters in the Violett-treated cage and the Control cage were moved to clean cages and maintained for an additional three days to determine whether airborne transmission of SARS-CoV-2 had occurred.

Individually ventilated hamster cages were modified to create a closed system where air would enter the cage holding infected hamsters and flow to peripheral cages holding naive hamsters. The Violett device was connected to a 3-inch diameter flexible tubing between the cages to allow air treatment (Figure 1). Air exchange within the system was about 60 changes per hour. A total of 27 hamsters were randomly divided into three groups of nine hamsters to allow three experimental replicates. Within each replicate three hamsters were randomly assigned to one of three groups: Infected, Violett-treated, or Control. Hamsters in the Infected group were infected intranasally with 1 × 10^4^ pfu SARS-CoV-2 virus (USA-WA1/2020) under sedation and co-housed in the central cage for 24 h (Figure 2). Prior to the addition of naïve hamsters, the sterilization devices were turned on for 15 min to ensure proper function. Next, with the devices continuously running, three naïve hamsters are introduced into each of the two peripheral cages (a total of six hamsters). The device remained on for a total of 48 hours during peak viral shedding by the infected hamsters in the central cage. At the completion of air sterilization, the hamsters in the peripheral cages were immediately moved to a clean hamster cage and maintained for three days to evaluate whether the hamsters became infected. Infected hamsters were removed from the central cage and terminated. All hamsters were humanely euthanized and necropsied at the end of the study.

### 2.4. Virus

SARS-CoV-2 propagation occurred in a BSL-3 laboratory setting. The virus(isolate USA-WA1/2020) was acquired through BEI Resources (product NR-52281) and amplified in Vero C1008 (Vero E6) cell culture. Vero E6 cells (ATCC CRL-1568) were cultured in Dulbecco’s modified Eagle’s medium (DMEM) supplemented with glucose, L-glutamine, sodium pyruvate, 5% fetal bovine serum (FBS) and antibiotics. Inoculation of Vero E6 cells with SARS-CoV-2 was carried out directly in DMEM containing 1% FBS. Supernatant harvested from infected cells 3–4 days after inoculation was clarified by centrifugation at 800× *g*, supplemented with FBS to 10% and frozen at −80 °C in aliquots. The virus titer was determined using a standard double overlay plaque assay [22]. 

### 2.5. Viral Challenge

SARS-CoV-2 virus was diluted in phosphate-buffered saline (PBS) to 1 × 10^4^ pfu/0.1 mL. The hamsters were first lightly anesthetized with 10 mg of ketamine hydrochloride and 1 mg of xylazine hydrochloride. The virus was administered to each hamster by pipetting 100 µL of the viral inoculum into the nares of each hamster (50 µL/nare). Virus back-titration was performed on Vero E6 cells immediately following inoculation. Hamsters were observed until fully recovered from anesthesia. 

### 2.6. Oropharyngeal Swabs

Oropharyngeal swabbing was performed on all hamsters prior to the live virus challenge, and then daily after viral challenge to evaluate viral shedding. The swabbing was performed by inserting a sterile cotton swab and rotating the swab in the oropharyngeal area of the mouth for about 5 s. Swabs were placed in viral transport media (DMEM containing 2% FBS) supplemented with antibiotics and an antifungal and then stored at −80 °C until further analysis. 

### 2.7. Clinical Observations

Body weights were recorded prior to viral exposure and then daily after exposure. Weights were recorded in grams. Clinical evaluations were performed daily during the study and included recording temperament, ocular discharge, nasal discharge, coughing/sneezing, dyspnea, lethargy, anorexia, and moribund.

### 2.8. Tissue Collection

Necropsies were performed three or five days after the live virus challenge to determine gross pathological changes in respiratory tissues. Tissues were collected for virus quantification and histopathology. For virus quantitation, portions of right cranial lung lobe, right caudal lung lobe, and nasal turbinate specimens from each hamster were weighed (100 mg per specimen) and homogenized in viral transport media with antibiotics and then frozen at −80 °C until the time of analysis. The tissue homogenates were briefly centrifuged and virus titers in the supernatant were determined using the plaque assay. For histopathology, portions of the left lobe, right middle lung lobe, and trachea were collected from each hamster. The tissues were placed in 10% neutral buffered formalin for seven days and then paraffin embedded and stained with hematoxylin and eosin using routine methods for histological examination.

### 2.9. Virus Titration

Plaque assays were used to quantify infectious virus in oropharyngeal swabs and tissues. Briefly, all samples were serially diluted 10-fold in DMEM+ 1% FBS supplemented with antibiotics. Confluent Vero E6 cell monolayers were grown in 6-well tissue culture plates. The growth media was removed from the cell monolayers and washed with PBS immediately prior to inoculation. Each well was inoculated with 0.1 mL of the appropri-ately diluted sample. The plates were rocked every 10–15 min for 45 min and then overlaid with 0.5% agarose in media with 7.5% bicarbonate and incubated for 1 day at 37 °C and 5% CO_2_. A second overlay with neutral red dye was added at 24 h and plaques were counted 48–-72 h post-plating. Viral titers are reported as the log_10_ pfu per swab or gram (g). Samples were considered negative for infectious virus if viral titers were below the limit of detection (LOD). The theoretical limit of detection was calculated using the following equation:

LOD = log [1/(N × V)]

where N is the number of replicates per sample at the lowest dilution tested and V is the volume used for viral enumeration (volume inoculated/well in mL). For oropharyngeal swabs the LOD was 10 pfu/swab or 1.0 log_10_ pfu/swab. For tissues the LOD was 100 pfu/g or 2.0 log_10_ pfu/g.

### 2.10. Histopathology

Histopathology was blindly interpreted by a board-certified veterinary pathologist. The H&E slides were evaluated for morphological evidence of inflammatory-mediated pathology in the lungs, and trachea, and the reduction or absence of pathological features was used as an indicator of protection against airborne SARS-CoV-2 exposure. Each hamster was assigned a score of 0–3 based on absent, mild, moderate, or severe manifestation, of pulmonary pathology including mural bronchial inflammation, neutrophilic bronchitis, consolidating pneumonia, and interstitial alveolar thickening. The sum of all scores was then provided for each hamster. H&E-stained lung tissue slides were scanned at 20 Å~magnification using an Olympus VS120 microscope, Hamamatsu ORCA-R2 camera, and Olympus VS-ASW 2.9 software at the Experimental Pathology Facility at Colorado State University.

### 2.11. Statistical Analysis

Group mean (n = 3) viral titers of swabs and tissues were analyzed using two-way ANOVA followed by a post hoc test to analyze differences between groups. In the case where samples reached the LOD, values were entered as 0 for statistical analysis. Data were considered significant if *p* < 0.05. Composite pathology scores (n = 3) were compared between groups using a one-way ANOVA followed by a post hoc test to analyze differences between groups. Analysis was performed using GraphPad Prism software version 9.2.0 (GraphPad Software, Inc, La Joia, CA, USA). 

## 3. Results

To evaluate the efficacy of UV-C light emitted by the Violett device against SARS-CoV-2, we developed an aerosol transmission system comprising modified hamster cages connected by tubing to direct airflow from cages holding infected hamsters to cages holding naïve hamsters (Figure 1). The air passed through the tubing and into the Violett device for sterilization over a 48-hour exposure period. Following exposure, the hamsters were maintained for an additional three days to determine whether SARS-CoV-2 transmission had occurred (Figure 2). 

### 3.1. Clinical Parameters

**Figure 3:**
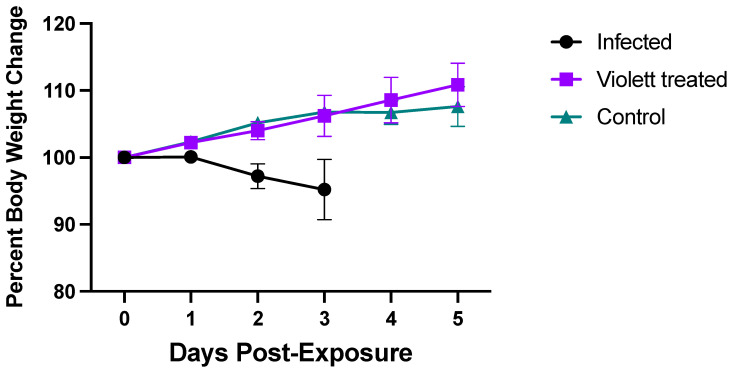
Body weight changes in hamsters after live virus exposure. Body weight changes in hamsters as a percentage after viral exposure. Body weights were monitored daily for 3 days in the Infected group (n = 9) and 5 days in the Control (n = 9) and Violett (n = 9) treatment groups. Data represented as the group mean +/− SD of the starting body weight.

None of the hamsters in any of the groups displayed signs of disease following viral exposure and were clinically normal. From the time of viral exposure to necropsy, the Infected group lost on average about 4.8% body weight over the 3-day period. Conversely, the Violett treated group and the Control group gained body weight (average of 10.8% and 7.8%, respectively) over the course of 5 days (Figure 3). There were no statistically significant difference between the groups.

### 3.2. Virus Titration

**Figure 4:**
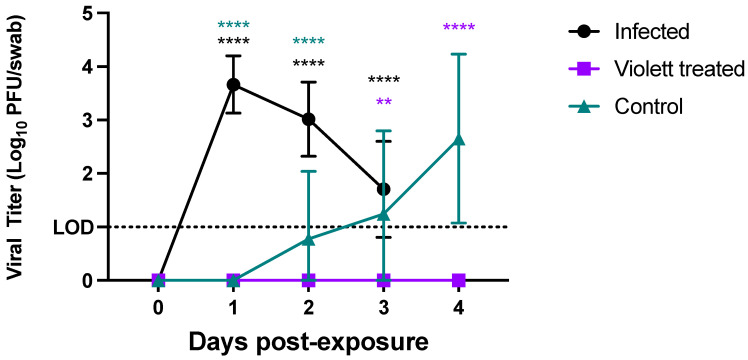
Viral shedding from oropharyngeal swabs. Viral loads in the oral pharyngeal swabs of hamster exposed to SARS-CoV-2. Viral titers were determined by plaque assay. Data points represent group mean +/− SD. LOD = Limit of detection denoted by horizontal dotted line. Asterisks in purple **** above bars indicate statistically statistically significant difference in viral titers between the Violett treated and the Control groups (**** *p* < 0.0001, ** *p* < 0.01). Asterisks in black **** above bars indicate statistically statistically significant difference in viral titers between the Violett treated and the Infected groups (**** *p* < 0.0001). Asterisks in teal **** above bars indicate statistically statistically significant difference in viral titers between the Control and the Infected groups (**** *p* < 0.0001).

We next investigated viral shedding in the oropharyngeal cavities of all the hamsters. Hamsters were swabbed prior to viral exposure and then daily until day 3 (in the Infected group) or day 4 (in the Violett treated and Control groups). Following the intranasal challenge, all hamsters in the Infected group had detectable infectious virus over the course of 3 days (except for one hamster on day 3) (Figure 4). Peak viral shedding was detected 1 day post-infection (DPI) with a mean titer of 3.66 log_10_ pfu/swab followed by a decline with a mean titer of 3.02 log_10_ pfu/swab 2 DPI and 1.92 log_10_ pfu/swab 3 DPI. Conversely, in the Control group, infectious virus was not detected in the swabs until 2 days post exposure to the Infected hamster group. The virus load increased to 2.65 log_10_ pfu/swab by day 4 indicating that airborne transmission did occur in the Control group. The variability detected in the swabs over the course of the infection can be attributed to the lower challenge dose and the gradual progression of disease following airborne exposure compared to the Infected group. Of most interest is the lack of detection of infectious virus in the oral cavities of hamsters in the Violett treated group at any time point. The difference in viral titers between the Violett treated group and the Control group were statistically significant 3 days (*p* = 0.0013) and 4 days post-exposure (*p* < 0.0001).

**Figure 5:**
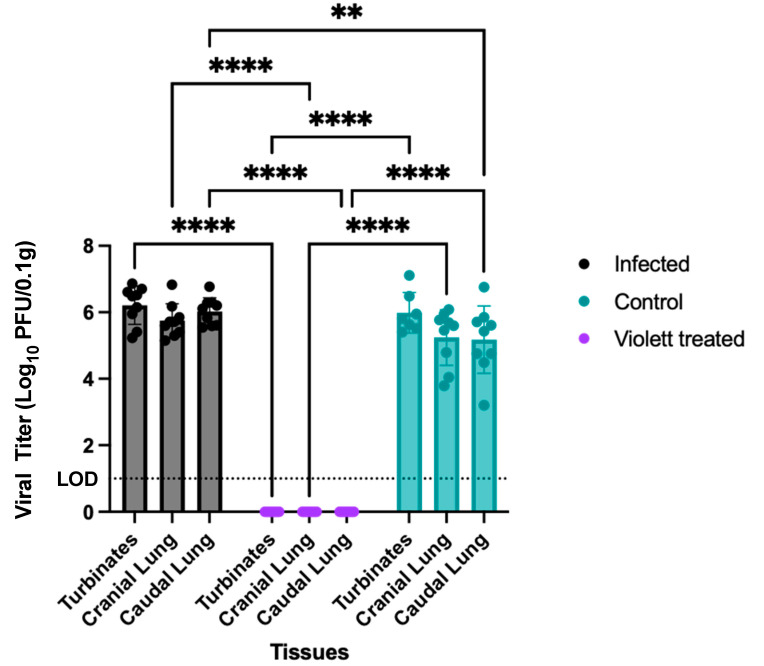
Viral burden of respiratory tract tissues. Viral replication in lower respiratory tract of hamster exposed to SARS-CoV-2. The presence of infectious virus was determined in turbinates, cranial lung lobe and caudal lung lobe of each hamster. Viral titers were determined by plaque assay. Data points represent group mean +/− SD. LOD = Limit of detection denoted by horizontal dotted line. Asterisks above bars indicate statistically statistically significant difference in viral titers (**** *p* < 0.0001, ** *p* < 0.01).

To confirm protection from airborne transmission in the Violett treated group, upper and lower respiratory tract tissues were tested for viral replication. Nasal turbinate and lung tissues from the Infected group were collected 3 days after intranasal exposure to SARS-CoV-2 while tissues from the Control and Violett treated groups were collected 5 days after exposure. Previous experimental infections with the SARS-CoV-2 Wuhan strain in a hamster model showed that viral replication between the cranial and caudal lung lobes was similar [23]. However, the cranial lobe is usually affected first during viral infection, while the caudal lobe can become affected later during the course of the disease [24], therefore, both lobes were tested for viral replication. In the Infected group, viral titers in the turbinates had a mean of 6.21 log_10_ pfu/0.1 g (Figure 5). Viral replication was slightly lower in the lungs, with a mean of 5.75 log_10_ pfu/0.1 g in the cranial lungs and 6.03 log_10_ pfu/0.1 g in the caudal lungs. In the Control group, viral titer mean was 5.99 log_10_ pfu/0.1 g in the turbinates, 5.24 log_10_ pfu/0.1 g in the cranial lungs, and 5.17 log_10_ pfu/0.1 g in the caudal lungs. As expected, the titers were lower than in the Infected group and more representative of natural infections. In contrast, no infectious virus was detected in respiratory tissues in the Violett treated group demonstrating that the Violett device prevented airborne transmission of SARS-CoV-2 to naïve hamsters.

### 3.3. Histopathology

**Figure 6:**
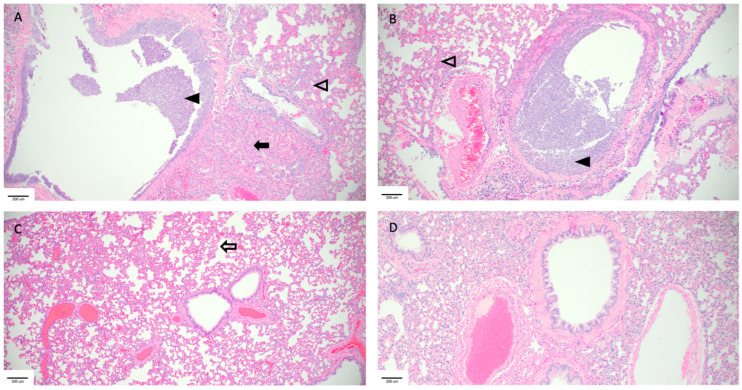
Pathological findings in hamster lungs. Representative histology of differences between intranasally infected (**A**), non-treated Control (**B**), Violett treated (**C**), and uninfected control (**D**). Image A was taken 3 days postchallenge and images (**B**,**C**) were taken 5 days post exposure. Infected hamsters (**A**,**B**) showed consolidating interstitial pneumonia (solid arrow), thickening of alveolar walls (open arrowhead), and accumulation of neutrophils within the bronchus lumen (solid arrowhead). There was minimal evidence of interstitial pneumonia (open arrow) and an absence of epithelial regeneration in the Violett-treated hamsters (**C**). Images taken at 10× magnification.

Hematoxylin and eosin (H&E) stained slides that included sections of lungs and trachea were reviewed for histopathological changes resulting from SARS-CoV-2 exposure (Figure 6). Hamsters intranasally challenged with SARS-CoV-2 (Infected group) demonstrated the most severe pulmonary pathology. Histopathological features of SARS-CoV-2 infection in this group included bronchial hyperplasia of secondary and tertiary bronchi that ranged from segmental cellular piling to diffuse epithelial thickening. Alveolar septa were thickened by mononuclear inflammatory cell infiltrates and centered in regions of pneumonia or diffusely throughout all septa. Inflammation was appreciated in the trachea. The Control group had the largest degree of variability in overt lesion burden but generally exhibited mild pathology compared to the infected group. Bronchi infiltrative inflammation, consolidated interstitial pneumonia, and thickening of alveolar walls were observed in most hamsters in the group. Similar to the Infected group, there was appreciable inflammation in the trachea of several hamsters. Minimal to no lesions were appreciated in the Violett treated group. Mild alveolar septa thickening was observed in some of the hamsters. However, the scoring was low and could be interpreted as being within normal histological limits.

**Figure 7:**
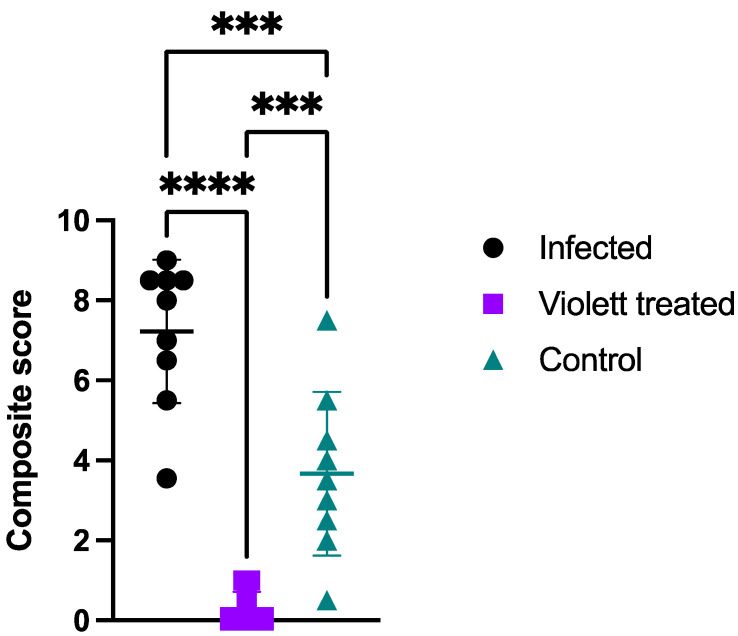
Composite histopathology score. Two sections of lung per hamster were evaluated. Sections were evaluated separately and then averaged to provide a mean score for that animal. Means of each parameter were added to provide a composite score. Data points represent mean +/− SD. Astricks above bars indicate statistically significant difference in composite score (**** *p* < 0.0001, *** *p* < 0.001).

Based on pathological scoring of lung tissues, the Violett treatment effectively prevented SARS-CoV-2 replication and pathology in respiratory tissues. The Infected group had a mean composite score of 7.2, the Control group had a mean composite score of 3.7, while the Violett-treated group had a significantly lower mean composite score of 0.28 (Figure 7). The differences in composite score were statistically significant between the Violetttreated and Infected groups (*p* < 0.0001) and the Violett treated and Control groups (*p* = 0.0004). All hamsters in the Infected group had appreciable lung lesions demonstrating the robustness of viral replication following intranasal challenge. The degree of lesions observed in the Control group was more variable compared to that observed in the Infected group; however, it is evident that airborne transmission did occur from the Infected group to the Control group. The Violett treatment prevented the development of COVID disease in the lungs of naïve hamsters.

## 4. Discussion

UV-C inactivation has gained widespread recognition for its efficacy against various pathogens, including viruses and bacteria [25]. With the rapid spread of SARS-CoV-2, environmental and structural changes became a valuable tool for reducing transmission in areas with high risk of exposure [26]. As we move out of a pandemic state, we continue to face uncertainty with newly emerging variants. These variants contain mutations that can enhance antibody evasion and increase transmissibility [27,28]. A multi-prong approach is needed to provide protective immunity and reduce exposure risk. Notably, environmental measures such as UV light treatment will be critical in mitigating viral transmission.

Our study adds to the growing body of evidence supporting the efficacy of UV-C light, specifically in inactivating SARS-CoV-2 in the air [18,29]. The fundamental mechanism of UV-C light relies on destroying the bonds within the genetic material of viruses and bacteria, a process that is pathogen-agnostic since nucleic acids are the fundamental building blocks of all life on Earth. While this study focused on transmission of SARS-CoV-2, this can be seen as a readout for any number of airborne diseases both known and still to be identified. Futhermore, the utility of UV-C light extends beyond simple viral inactivation; it can play a pivitol role in mitigating the impacts of disease like COVID-19. This has significant implications for patient safety, especially for those with weakened immune systems who are at higher risk of severe COVID disease [30]. This means protective measures that use UV-C light, such as the Violett device, circumvents the adverse effects associated with SARS-CoV-2 infections such as inflammation or cytokine storm syndrome [8,31]. The application of this kind of technology not only addresses current healthcare challenges but it is also future-proofing our systems against the rise of new diseases and variants. 

Previous studies have corroborated the use of UV-C light in preventing airborne SARS-CoV-2 transmission in a hamster model. Hamsters are a well-established animal model for SARS-CoV-2 airborne transmission studies [32,33,34,35]. Bowen et al. demonstrated protection from airborne transmission with the use of a UV-C device over a 48 hour period of exposure [36]. Individual hamsters housed in UV-C air treated cages did not have viral replication three days after exposure. To evaluate efficacy against VOCs, Fischer et al. tested both the WA-1 and the Delta strain against a UV-C device [20]. In the study two, naïve hamsters were exposed to aerosols from infected hamsters for a total of four hours and then orally swabbed for three days and maintained till 14 days post exposure to determine whether transmission occurred. All treated hamsters were protected from airborne transmission of both the WA-1 and Delta strains. The present work further confirms the effectiveness of UV-C in preventing airborne transmission of SARS-CoV-2. We demonstrated the robustness of the Violett device in preventing transmission during peak viral shedding in a group setting comprising susceptible hosts.

The length of exposure in these studies is of particular note. This more accurately mimics a shared room in a medical setting (e.g., hospital) and indicates a level of protection that would be meaningful in a real-world setting with elevated risk from prolonged exposure. In the Violett device all UV-C sources are fully contained so that it can be operated while a room is occupied. This differentiates the device from other technologies that require rooms to be cleared so that UV-C light can be shone outward onto surfaces. Since the Violett device allows for continuous cleaning of an occupied room it provides more comprehensive protection to occupants. 

The risk of prolonged viral exposure extends beyond the medical setting as well. The Violett device would provide added safety to children in schools, patients in waiting rooms, and workers in offices. The portable nature of the Violett device means that it can be flexibly used in a variety of settings where large numbers of people gather for extended periods. In all these settings, proper placement of a Violett device in the room heightens the efficacy and protection provided. While the Violett device uses a fan to push air through the HEPA filter and UV-C light it is also helpful to place the device in a central location to ensure even treatment of air in the entire space. In hospitals this can be between patients beds, for example. The 360-degree air inlet of the device allows air to be drawn in from all sides of the device hence the most efficacious placements allow a few inches of clearance on all sides of the device.

There is nothing more fundamental to our health and well-being than the air we breathe. The global pandemic has shown how devasting airborne diseases can be to both healthy individuals, but especially to our most vulnerable populations. Beyond the risk of disease and the associated morbidity, the social and emotional toll of isolation over the last few years has been extreme. The spike in mental health issues and depression that is being seen as a direct result of this pandemic is just beginning to be documented, but the numbers are alarming. The picture is even more severe when you look at higher-risk groups such as older adults or immune compromised patients. This isolation leads to lower compliance with medication and health directives which can cause severe medical complications [37]. The Violett UV-C sterilization device offers a solution for creating safer environments for both healthcare workers and patients, presenting a crucial step toward mitigating health threats caused by airborne pathogens. 

## 5. Patents

The USPTO application number for the Violett technology is 17574159.

## Data Availability

Data are contained within the article.
